# Supplementary material for: Lipid droplet and peroxisome biogenesis occur at the same ER subdomains
Source: Nat Commun. 2018 Jul 27;9:2940. doi: 10.1038/s41467-018-05277-3 (PMC6063926; doi:10.1038/s41467-018-05277-3)
Supplement: Supplementary file 1 — Supplementary Information [file 41467_2018_5277_MOESM1_ESM.pdf]

Lipid droplet and peroxisome biogenesis occur at the same ER subdomains.

Joshi et al.

Supplementary Figure 1

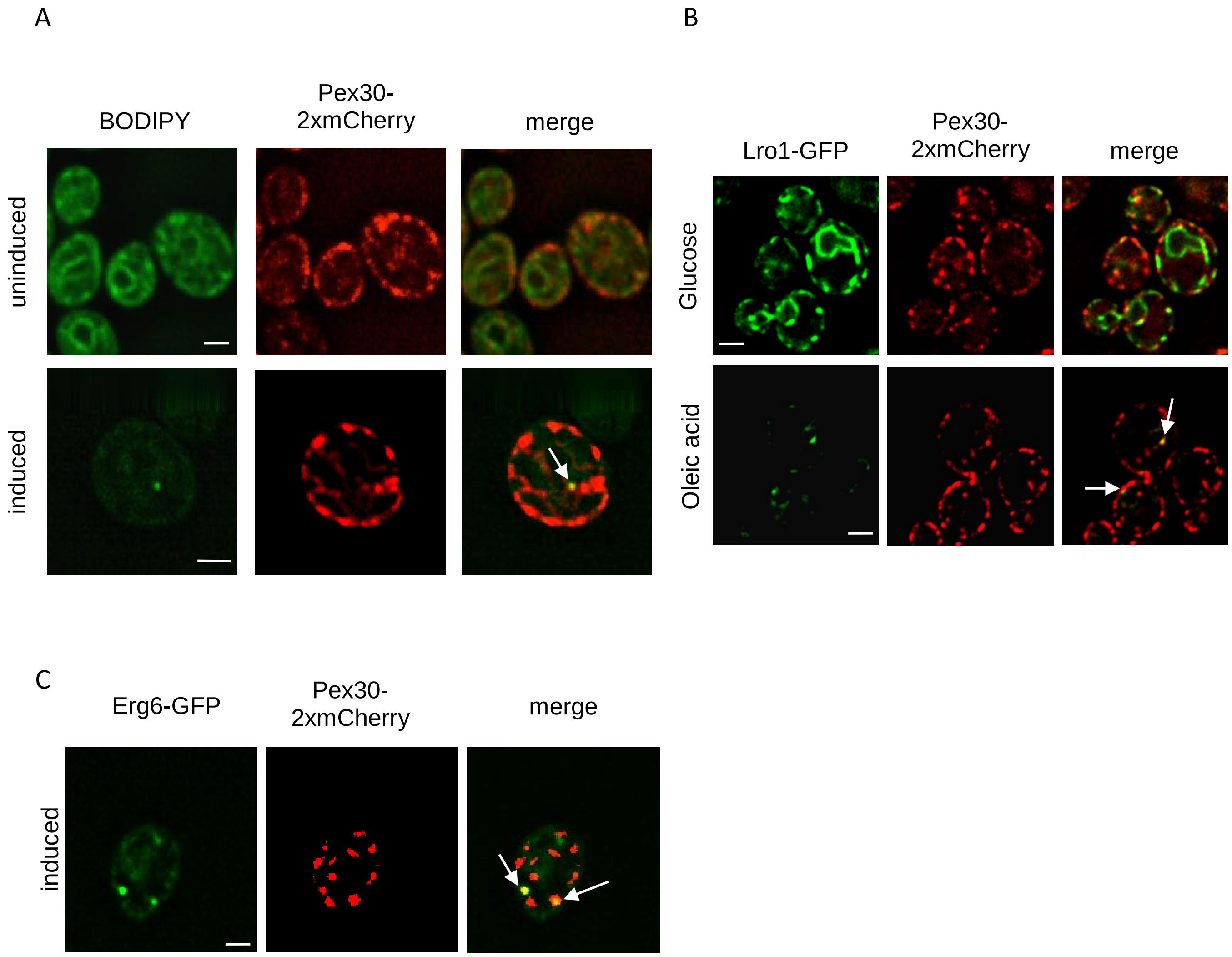

Supplementary Figure 1. Data associated with Fig. 1.

A) *GAL1-LRO1* 3Δ cells expressing endogenously tagged Pex30-2xmCherry were stained with BODIPY and visualized after growing in a medium containing raffinose (uninduced) or 30 minutes after galactose addition (induced). White arrows indicate colocalization of Erg6-GFP and Pex30-2xmCherry puncta. Bar = 3μm.

B) Same as Fig. 1C except cells are expressing endogenously tagged Pex30-2xmCherry and Lro1-GFP from a plasmid. Bar = 3μm.

C) Same as Fig. 1A except images were acquired by focusing on periphery of cells. Bar = 3μm.

Supplementary Figure 2

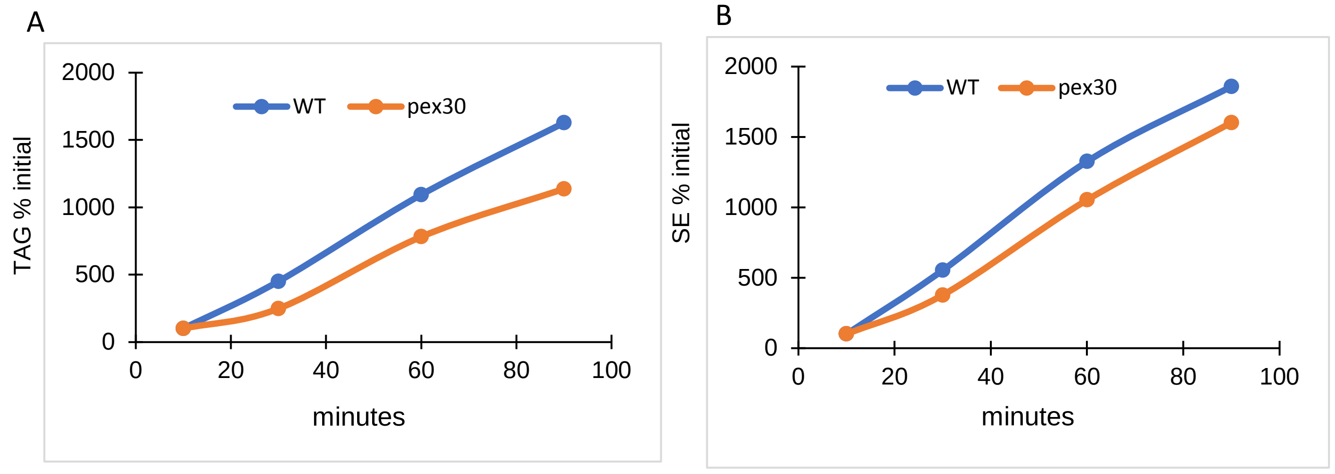

Supplementary Figure 2. Data associated with Fig. 3. Relative rate of neutral lipid synthesis in WT and *pex30*Δ cells. Cells were grown to mid-logarithmic growth phase in SC medium. The relative amount of TAG (A) and SE (B) were determined at the indicated times after addition of [^{3}H]acetate. Mean values of the percent initial levels of TAG (A) and SE (B) from two independent experiments are shown.

Supplementary Figure 3

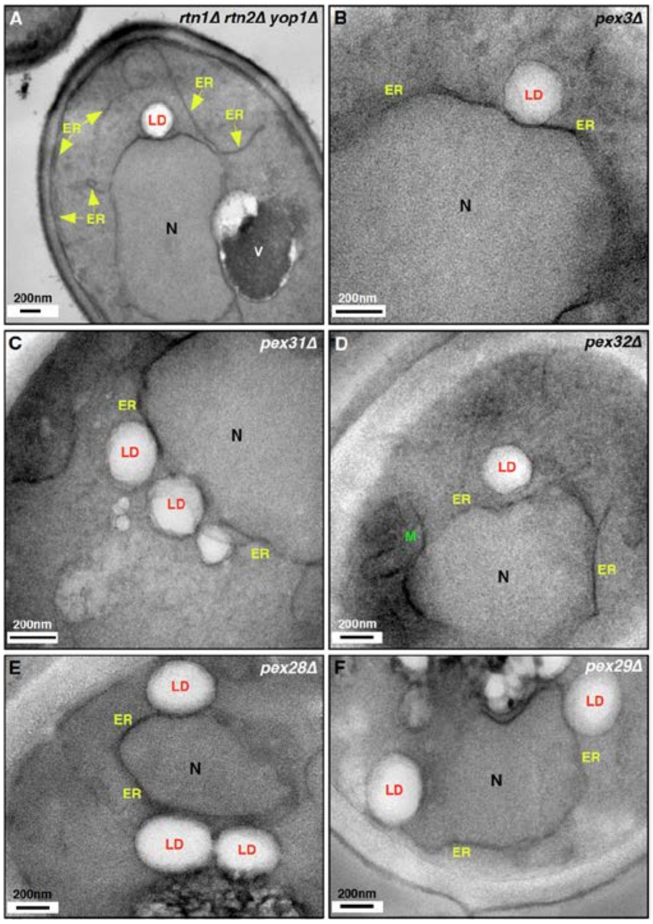

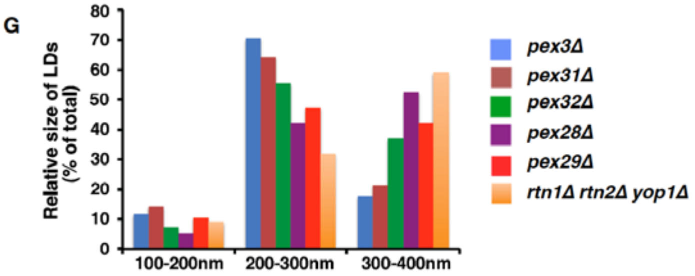

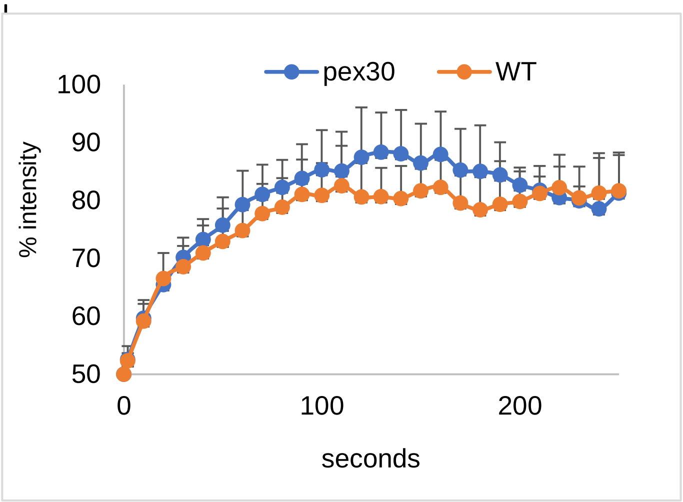

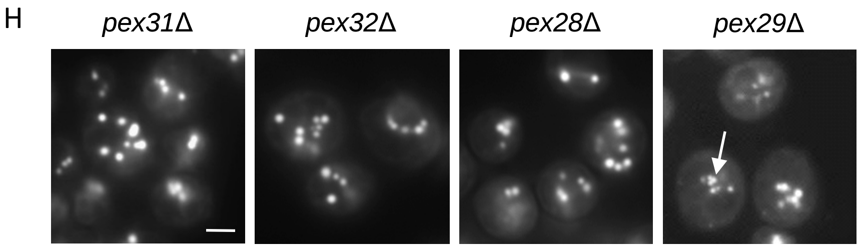

Supplementary Figure 3. Data associated with Fig. 3.

A-F) Cells growing in SC were fixed and visualized by EM. Yellow arrows indicate ER. V; vacuole, N; Nucleus, LD; Lipid droplets.

G) Quantification of LD diameter in experiments shown in A-F, n= 20 cells.

H) Cells were grown to early stationary growth phase in SC and stained with BODIPY to visualize LDs. Stacks of 10 images with a step size of 0.2µm were taken; images from a single plane are shown. Arrows indicate clustered LDs. Bar = 3µm.

I) FRAP of Dga1-GFP on LDs. Shown are the averages of the recovery of Dga1-GFP percent fluorescence intensity on LDs in wild-type and *pex30*Δ. Mean ± S.E (n=10).

No	Hit	Prob	E-value	2Sim	COLs	MCTP2 region	Length of Hit	Region of Hit
1	Pex29p	99.8	8.8E-21	22.3	180	40-223	554	111-307
2	Pex28p	99.8	1.1E-20	21.3	189	40-231	579	197-431
3	Pex30p	98.9	4.2E-11	17.4	146	48-223	523	64-218
4	Pex31p	98.5	3.0E-09	14.8	145	48-223	462	56-210
5	Pex32p	97.6	5.7E-06	16.5	146	48-223	413	31-212
6	Rtn1p	97.0	9.8E-05	13.8	137	69-227	295	17-164
7	Rtn2p	96.6	0.00036	13.1	148	65-228	393	23-175

Supplementary Figure 4. Data associated with Fig. 5. HHpred analysis of MCTP2 C-terminus homologs in *S. cerevisiae*. Close homologues in yeast of the C-terminal 272 residues of MCTP2 (607-878) were identified by HHpred as having probability of shared structure (“Prob”) >95%. For each hit, HHpred also returns other values: the statistical expectation of achieving the hit in terms of sequence alone (“E-value”); the secondary structural similarity, which is independent of sequence homology (2Ssim); and the number of columns aligned between query and target (COLs); as well as the regions of MCTP2 and each hit that overlap and the length of each hit.

Supplementary Figure 5

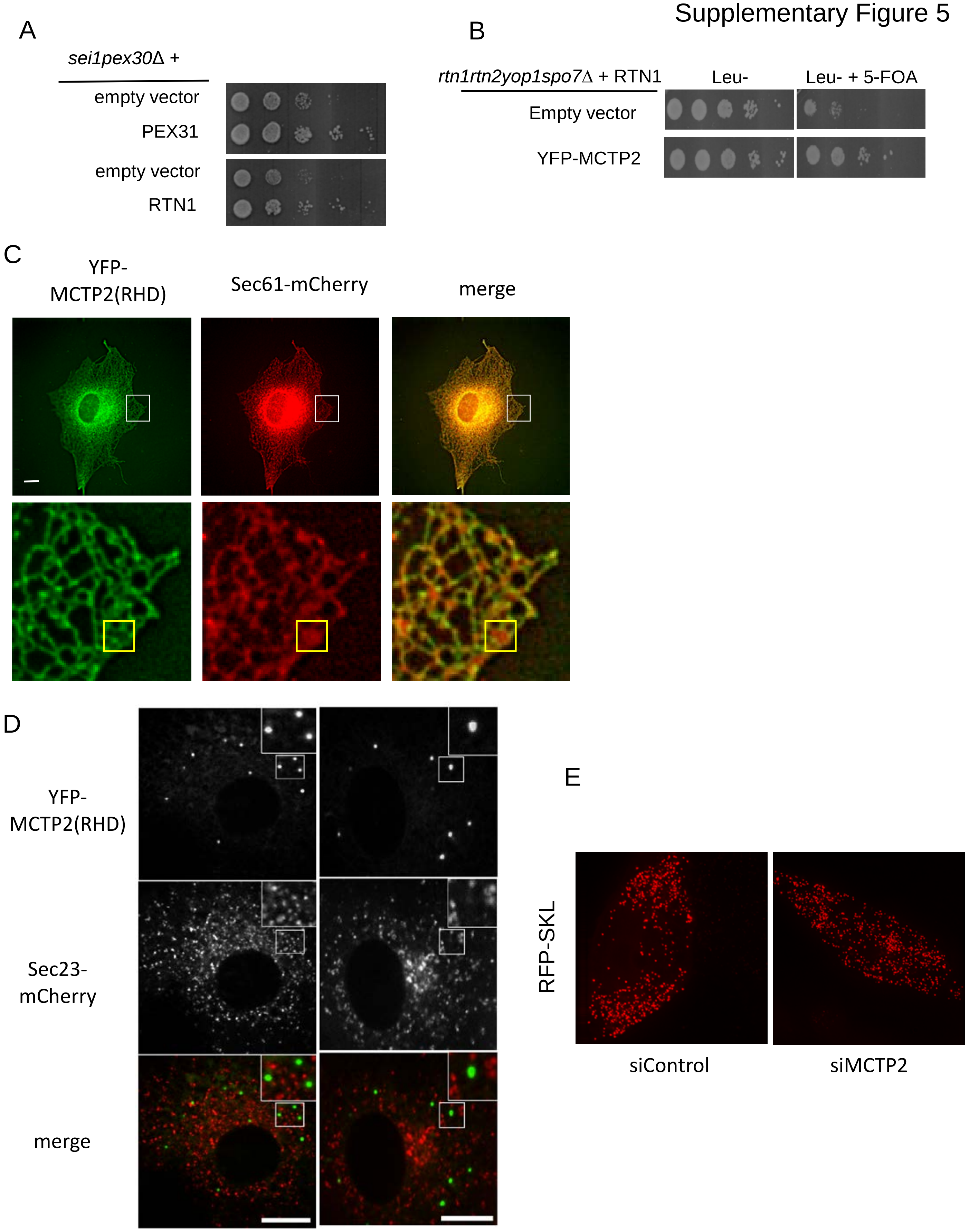

Supplementary Figure 5. Data associated with Fig. 5.

A) Same as Fig 5B.

B) Cells lacking Rtn1, Rtn2, Yop1, and Spo7 (*rtn1rtn2yop1spo7*Δ) are not viable unless they contain a plasmid expressing an RHD-containing protein. The *rtn1rtn2yop1spo7*Δ cells were complemented with a plasmid containing *RTN1* and *URA3*, which can be counter selected with 5-Fluoroorotic Acid (5-FOA). This strain containing an empty vector or a plasmid expressing YFP-MCTP2 (RHD) was grown to mid-logarithmic growth phase, serially diluted, spotted on to SC plates with or without 5-FOA, and incubated at 30°C for 3 days.

C) Same as Fig. 5D but showing a cell expressing YFP-MCTP2 (RHD) at a high level. Region in white box shown in higher magnification in lower panels. Yellow box shows ER sheet with YFP-MCTP2 (RHD) at edges. Bar = 5μm.

D) COS7 cells expressing YFP-MCTP2(RHD) and Sec23-mCherry. Region in white box shows higher magnification.

E) HeLa cells expressing the peroxisome marker RFP-SKL and treated with siRNA.

Supplementary Figure 6

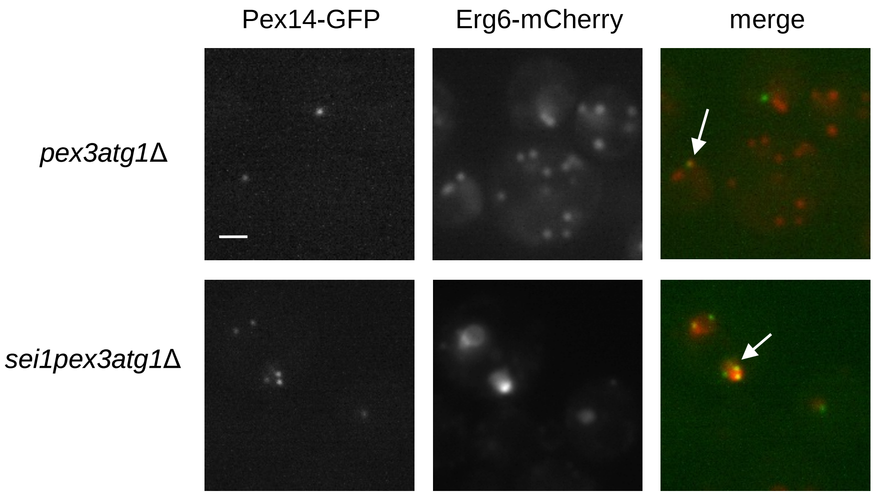

Supplementary Figure 6. Data associated with Fig. 7B.

Cells with the indicated genotypes were grown and visualized as in Fig. 7B, bar = 3μm.

Supplementary Table 1: Strain list		
**Strain**	**Genotype**	**Source**
BY4741	MATa *his3Δ1 leu2Δ0 met15Δ0 ura3Δ0*	Laboratory collection
BY4742	MATalpha *his3Δ1 leu2Δ0 lys2Δ5 ura3Δ0*	Laboratory collection
AJY2	BY474 MAT? *rtn1::KanMx6 rtn2::KanMx6 yop1::KanMx6 spo7::NatMx6 his3Δ1 leu2Δ0 ura3Δ0* Ycplac33-RTN1	Joshi et al. JCB, 2016
CVY458	W303 MAT? *rtn1::KanMx6, rtn2::KanMx6, yop1::KanMx6, ss-RFF-HDEL-TRP1*	Laboratory collection
CVY215	W303 MAT? *leu2-3, -112, his 3-11,-15, trp1-1, ura3-1, ade2-1, can1-100*, ss-RFF-HDEL-TRP1	Laboratory collection
AJY523	BY4742 MATalpha *his3Δ1 leu2Δ0 lys2Δ5 ura3Δ0 are1::KanMX are2::KanMX GAL-LRO1::TRP1 dga1::loxP PEX30-2xmCherry-URA3 ERG6-GFP-HIS3*	This study
AJY465	BY474 MAT? *PEX30-2xmCherry-URA3 ERG6-GFP-HIS3 leu2Δ0*	This study
AJY420	BY474 MAT? *PEX30-2xmCherry-URA3 NEM1-GFP-HIS3 leu2Δ0*	This study
AJY500	BY474 MAT? *PEX30-2xmCherry-URA3 SEI1-GFP-HIS3 leu2Δ0*	This study
AJY388	BY474 MAT? *PEX30-2xmCherry-URA3 his3Δ1 leu2Δ0*	Joshi et al. JCB, 2016
AJY343	BY474 MAT*? pex30::KanMx6 his3Δ1 leu2Δ0 ura3Δ0*	This study
AJY471	BY4742 MATalpha *pex31::NatMx6 his3Δ1 leu2Δ0 ura3Δ0 lys2Δ5*	This study
AJY557	BY474? MAT? *pex32::HIS3 leu2Δ0 ura3Δ0*	This study
AJY64	BY4742 MATalpha *pex28:hyg his3Δ1 leu2Δ0 ura3Δ0 lys2Δ5*	This study
AJY67	BY4741 MATa *pex29::KanMx6 his3Δ1 leu2Δ0 ura3Δ0 met15Δ0*	This study
AJY28	BY4741 MATa *spo7::NatMx6 his3Δ1 leu2Δ0 ura3Δ0 met15Δ0*	Joshi et al. JCB, 2016
AJY199	BY474 MAT? *rtn1::KanMx6 rtn2::KanMx6 yop1::KanMx6 his3Δ1 leu2Δ0 ura3Δ0*	This study
AJY342	BY474 MAT? *sei1::KanMx6 his3Δ1 leu2Δ0 ura3Δ0*	This study
AJY345	BY474 MAT? *sei1::KanMx6 pex30::KanMx6 his3Δ1 leu2Δ0 ura3Δ0*	This study
AJY574	BY474 MAT? *sei1::KanMx6 his3Δ1 leu2Δ0 ura3Δ0 PEX30-HA-HIS3*	This study
AJY577	BY474 MAT? *sei1::KanMx6 his3Δ1 leu2Δ0 ura3Δ0 PEX30 (1-235 amino acid residues)-HA-HIS3*	This study
AJY421	BY474 MAT? *sei1::KanMx6 PEX30-2xmCherry-URA3 NEM1-GFP-HIS3 leu2Δ0*	This study
AJY613	BY474 MAT? *pex3::NatMx6 atg1::hyg PEX30-2xmCherry-URA3 PEX14-YFP-KanMx6 his3Δ1* Yeplac181-ERG6-BFP	This study
AJY522	BY4742 MATalpha *his3Δ1 leu2Δ0 lys2Δ5 ura3Δ0 are1::KanMX are2::KanMX trp1::URA3 GAL-LRO1::TRP1 dga1::loxP PEX30-2xmCherry-URA3 his3Δ1*	This study
AJY422	BY474 MAT? *pex3::NatMx6 atg1::hyg PEX14-GFP-URA3 ERG6-mCherry-HIS3 leu2Δ0*	This study
AJY423	BY474 MAT? *sei1::KanMx6 pex3::NatMx6 atg1::hyg PEX14-GFP-URA3 ERG6-mCherry-HIS3 leu2Δ0*	This study
AJY639	BY474 MAT? *PEX30-2xmCherry-URA3 SEC13-GFP-HIS3 leu2Δ0*	This study
AJY640	BY4742 MATalpha *his3Δ1 leu2Δ0 lys2Δ5 are1::KanMX are2::KanMX GAL-LRO1::TRP1 dga1::loxP pex30::CaURA3*	This study
AJY634	BY474 MAT? *PEX30-2xmCherry-URA3 PEX14-YFP-KanMx6 his3Δ1* Yeplac181-ERG6-BFP	This study

Supplementary Table 2: Plasmid list			
**Name**	**Plasmid**	**Description**	**Source**
pAJ181	YEplac181-proADH1-(ER-DAG sensor)-tCYC1	PKD-GFP-UBC6 (ER-DAG sensor) expressed under ADH1 promoter	This study
pAJ164	YEplac181-DGA1-GFP	DGA1-GFP expressed under DGA1 promoter	This study
pAJ168	YCplac111-DGA1-GFP	DGA1-GFP expressed under DGA1 promoter	This study
pAT121	YCplac111-SEC63-mCherry	SEC63-mCherry expressed under Sec63 promoter	Laboratory collection
pAJ141	YCplac181-proRTN1-YFP-MCTP2	YFP-MCTP2 expressed under RTN1 promoter	This study
pAJ48	YEplac181-proRTN1-PEX30	PEX30 expressed under RTN1 promoter	This study
pCV47	YCplac33-RTN1	RTN1 expressed under RTN1 promoter	Laboratory collection
pAJ195	YCplac181-ERG6-BFP	ERG6-BFP expressed under ERG6 promoter	This study
pAJ171	YCplac111-LRO1-GFP	LRO1-GFP expressed under LRO1 promoter	This study
pAJ212	C1-EGFP-MCTP2	GFP-MCTP2 expressed under CMV promoter	This study

Supplementary Methods:

Fluorescence recovery after photobleaching (FRAP)

Cells were transformed with plasmid Yep181-Dga1-GFP. FRAP experiments were conducted on a Zeiss LSM 780 laser scanning inverted microscope using a Plan Apo 100x/1.4 oil objective with argon laser line 488 nm. LSM software ZEN was used for image acquisition and analysis. Images, were taken pre-bleaching followed by photo bleaching a region of interest (14 x 14 pixels) at 100% laser power until the intensity was 50% the original intensity. After photo bleaching images were taken every 10 seconds at room temperature for 240 seconds.
