# Supplementary material for: Lipid droplet and peroxisome biogenesis occur at the same ER subdomains
Source: Nat Commun. 2018 Jul 27;9:2940. doi: 10.1038/s41467-018-05277-3 (PMC6063926; doi:10.1038/s41467-018-05277-3)
Supplement: Supplementary file 3 — Description of Additional Supplementary Files [file 41467_2018_5277_MOESM3_ESM.pdf]

Description of Additional Supplementary Files

File Name: Supplementary Movie 1
Description: **YFP-MCTP2 is stably associated with ER subdomains.** Time-lapse images of COS7 cells co-transfected with plasmids expressing Sec61-mCherry and YFP-MCTP2 (RHD). Stacks of 20 images with a step size of 0.3μm were taken for 10 minutes with a time interval of 60 seconds and deconvolved; images from a single plane are shown.

File Name: Supplementary Movie 2
Description: **Peroxisomes associating transiently with ER subdomains containing YFP-MCTP2 (RHD) and LiveDrop-mCherry.** Time-lapse images of COS7 cells co-transfected with plasmids expressing Sec61-mCherry, CFP-SKL and YFP-MCTP2 (RHD). Stacks of 20 images with a step size of 0.3μm were taken for 2.5 minutes with a time interval of 12 seconds and deconvolved; images from a single plane are shown.

File Name: Supplementary Movie 3
Description: **Peroxisomes associating stably with ER subdomains containing YFP-MCTP2 (RHD) and LiveDrop-mCherry.** Time-lapse images of COS7 cells co-transfected with plasmids expressing Sec61-mCherry, CFP-SKL and YFP-MCTP2 (RHD). Stacks of 20 images with a step size of 0.3μm were taken for 2.5 minutes with a time interval of 12 seconds and deconvolved; images from a single plane are shown.
